# Supplementary material for: Causal interplay between lactose intolerance and gut microbiota: a combined bidirectional Mendelian randomization and in vivo validation study
Source: Front Nutr. 2026 Jun 1;13:1803337. doi: 10.3389/fnut.2026.1803337 (PMC13265576; doi:10.3389/fnut.2026.1803337)
Supplement: Supplementary file 1 [file Data_Sheet_1.zip › supplementary materials/Forward/leave one out cross validation/ebi-a-GCST90027766.finngen_R12_E4_LACTONAS.pdf]

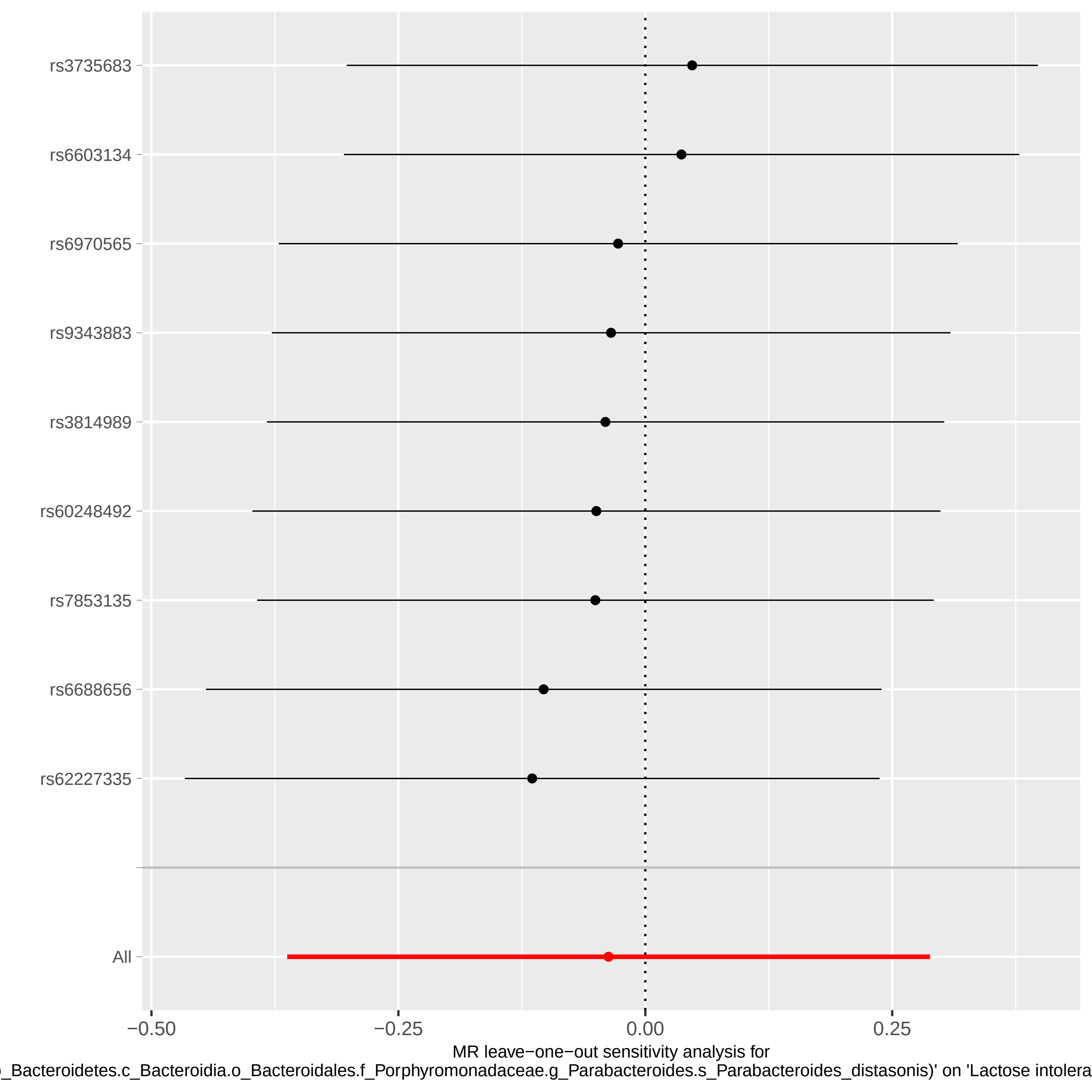
rs3735683
rs6603134
rs6970565
rs9343883
rs3814989
rs60248492
rs7853135
rs6688656
rs62227335
All
−0.50
−0.25
0.00
0.25
MR leave−one−out sensitivity analysis for
_Bacteroidetes.c_Bacteroidia.o_Bacteroidales.f_Porphyromonadaceae.g_Parabacteroides.s_Parabacteroides_distasonis)' on 'Lactose intolera
